# Integrating Life-Review and Drama Therapy for Community-Dwelling Older Adults: An Evidence-Based Model

**DOI:** 10.1093/geroni/igab046.2173

**Published:** 2021-12-17

**Authors:** Shoshi Keisari

**Affiliations:** University of Haifa, Department of Gerontology, Faculty of Social Welfare and Health sciences, Mount Carmel, HaZafon, Israel

## Abstract

Drama therapy is a widely acknowledged way to explore life-stories in late life. This presentation will describe a new model for creative interventions, based on the results of four studies that provide multiple perspectives on the integration of life-review and drama therapy for community dwelling older adults. The results of two quantitative studies (n=55, aged 62-93; n=78, aged 63-96) suggest that the drama therapy interventions have robust therapeutic potential to enhance mental health while aging. The findings of two qualitative studies with therapists (n=8), participants (n=27; aged 63-96) and staff (n=13) provide a better understanding of the process, and support the mechanisms that lead to positive effects on mental health. Combining the results yielded a multidimensional model which points to three potential transformative routes: the evolution of the life-story, the evolution of improvised dramatic expression, and the expansion of social engagement.

